# Possibilities of the Development of Edible Insect-Based Foods in Europe

**DOI:** 10.3390/foods10040766

**Published:** 2021-04-03

**Authors:** Magdalena Skotnicka, Kaja Karwowska, Filip Kłobukowski, Aleksandra Borkowska, Magdalena Pieszko

**Affiliations:** 1 Departament of Commodity Science, Faculty of Health Sciences, Medical University of Gdańsk, 80-210 Gdańsk, Poland; kaja.karwowska@gumed.edu.pl (K.K.); fklobukowski@gumed.edu.pl (F.K.); aleksandra.borkowska@gumed.edu.pl (A.B.); 2 Departament of Clinical Nutrition and Dietetics, Faculty of Health Sciences, Medical University of Gdańsk, 80-210 Gdańsk, Poland; magdalena.pieszko@gumed.edu.pl

**Keywords:** insects, mealworms, grasshopper, locust, cricket, buffalo worms

## Abstract

All over the world, a large proportion of the population consume insects as part of their diet. In Western countries, however, the consumption of insects is perceived as a negative phenomenon. The consumption of insects worldwide can be considered in two ways: on the one hand, as a source of protein in countries affected by hunger, while, on the other, as an alternative protein in highly-developed regions, in response to the need for implementing policies of sustainable development. This review focused on both the regulations concerning the production and marketing of insects in Europe and the characteristics of edible insects that are most likely to establish a presence on the European market. The paper indicates numerous advantages of the consumption of insects, not only as a valuable source of protein but also as a raw material rich in valuable fatty acids, vitamins, and mineral salts. Attention was paid to the functional properties of proteins derived from insects, and to the possibility for using them in the production of functional food. The study also addresses the hazards which undoubtedly contribute to the mistrust and lowered acceptance of European consumers and points to the potential gaps in the knowledge concerning the breeding conditions, raw material processing and health safety. This set of analyzed data allows us to look optimistically at the possibilities for the development of edible insect-based foods, particularly in Europe.

## 1. Introduction

Edible insects have been a part of human diets since antiquity, but a degree of distaste for their consumption exists in some regions of the world [1,2,3]. To this day, the prospect of eating insects is regarded as a new phenomenon for Western consumers.

Even a few years ago, in the majority of Western countries, one could find only a few examples of the use of insects in the diet, mainly by combining them with other meals and preparation methods. Such an approach was considered to be more of a novelty than a need or actual demand, as these products have been created only for specific events or occasions to arouse curiosity in people [4,5].

On the other hand, in view of the growing world population, increasingly demanding consumers and the decreasing availability of agricultural areas, there is a strong need to search for an alternative to conventional protein sources, all the more so that the animal production is among the main causes of climate change. Within the framework of sustainable development, it would be appropriate to consider the introduction of insect-based products into the European daily diet.

Insects are a significant biological resource which is still not fully exploited, especially in Europe. There are many insect species that could be a valuable and safe food ingredient. Insect bodies are rich in protein, amino acids, fat, carbohydrates, various vitamins and trace elements. In recent years, a much greater variety of insect-based products have been offered in Western countries. There is growing interest among entrepreneurs in this new food ingredient in the form of crisps, energy bars and other functional food products.

Insects can be acquired in three ways: gathering wild insects in various parts of the world, partial domestication, and industrial farming. Currently, 92% of products are obtained from traditional gathering, while only 2% are from industrial production [6]. However, having considered the development of this branch of industry, only the latter method has potential since it ensures stable supply and health safety, particularly in the European market. The market of food based on edible insects in Europe is developing very dynamically and many companies have noticed its potential. The Insect Food Business Operators (iFBOs) estimate that out of 500 tons of edible insects in 2019, the market will expand to 260,000 tons by 2030. As regards the consumption worldwide, the most often consumed species include beetles *Coleoptera* (31%), followed by *Lepidoptera* caterpillars (18%), honey bees, wasps, and ants *Hymenoptera* (14%), grasshoppers, locusts and crickets *Orthoptera* (13%). The remaining species include *Hemiptera*, *Isoptera*, *Odonata* and *Diptera* which are decidedly less likely to function within the commercial space [7]. Most edible insects are gathered in the wild and the concept of breeding them for food is relatively new. Despite the many benefits associated with introducing insects on the food market, it seems that the biggest obstacle to the development of this segment in Europe is the way it is perceived by potential consumers and the lack of developed culinary practices in this area. Therefore, educational and marketing activities should be carried out in parallel with legislative work and the safety assessment of insect-based products. For this reason, the aim of this study was to organize knowledge about edible insects, present the current legal status in the European Union and present the possibilities of developing insect-based food in Europe.

The work presents the current situation on the food market in the EU countries and the possible perspective of changes in the edible insect sector. The paper indicates potential threats and gaps in knowledge regarding breeding, health safety and barriers related to the introduction of insects to the European market. In the European Union, work is underway on the conditions for the cultivation of edible insects on an industrial scale and on risk assessment of selected insects. The creation of appropriate legal conditions for the development of entomophagy in Europe is a strong foundation for further changes.

## 2. Regulations Concerning Insect Production and Sales in the World and in Europe

Since 2003, the UN Food and Agriculture Organization (FAO) has been addressing the subject of insects and carrying out activities in many countries worldwide, including the collection of information on insects. In addition, the FAO participates in local projects associated with insect farming for consumption purposes. In countries with a long-standing tradition of insect consumption, there are appropriate regulations in place which enable production. However, in the countries where entomophagy is a new trend, there is a lack of appropriate legislation which hampers the development of this market [8]. The likelihood that insects could become more available on the European market as food has recently become possible thanks to the full application of a new regulatory framework for novel foods. The European insect production sector was initially based mainly on small- to medium-sized start-ups which have undertaken insect breeding for zoological gardens for biocontrol purposes or the production of animal feed [9]. Following the FAO report published in 2009, which demonstrated that the wide-scale production of insects may contribute to the reduction in hunger worldwide and limit the intensive rearing of slaughter animals [10], new insect-breeding enterprises were established in highly developed countries and research into the potential use of insects for consumption was launched.

Until 2018, the concept of edible insects as a food product did not exist in the European legal order. Their consumption was not banned by European legislation either, therefore each country could decide independently in this regard. The production of insects for consumption was possible under the general principles of food law (Regulation (EC) No 178/2002). In accordance with the precautionary principle, it was necessary to identify potential hazards posed by novel foods, to conduct the risk assessment, and to develop temporary risk management measures. Since 1997, it has also been possible to apply the procedure for the introduction of insects as a novel food or novel food ingredients under Regulation (EC) No. 258/97 of the European Parliament and of the Council of 27 January 1997 [11,12,13].

Currently, under the new provisions of Regulation (EU) No. 2015/2283, products placed on the market before 2018 under the previously applicable rules shall be reported to the European Commission as a “novel food” or a “traditional food from a third country” and, until an opinion is issued, can continue to be marketed [14]. The products introduced under the previous requirements (Regulation (EC) No. 258/97) are automatically qualified as a novel food. However, due to certain inaccuracies in provisions of the old regulations, doubts have arisen as to whether or not whole insects should be recognized as falling within the scope of the Regulation. This problem returned following the introduction of new restrictions. Certain European countries considered that whole insects should become subject to previous requirements for novel foods, and suspended or banned their sale on the domestic market. However, other countries, such as Italy, Portugal or Sweden, considered that whole insects and derived products should be recognized as a novel food pursuant to Regulation (EC) No. 258/97, and therefore refused to comply with the transitional measures set out in Regulation (EU) No. 2015/2283 [8,15].

The list of insects approved for consumption along with their characteristics, quality requirements, a list of food categories in which they can be used, and the maximum levels to be used in individual groups of products should be included in the EU’s list of novel foods. No insect has been included in the document drawn up on 20 December 2017 because, in the first instance, an individual business entity must apply to the European Commission for permission to place a specific insect species on the market in the European Union. The Commission charges the European Food Safety Authority (EFSA) with the task of issuing an opinion concerning the safety of consumption and the conditions for the production of food described in the application. New food is included in the list and can be marketed only after authorization [14,16,17].

In November 2020, the European Food Safety Authority finished considering the application for the recognition of mealworm larvae as novel food (EFSA-Q-2018-00262). According to the published opinion, mealworm larvae can be used as whole, dried as snack products and ground, powdered in various other food products: baked goods, energy bars, pasta (ON-6343). Provided that the European Commission’s Health Directorate General confirms this opinion, it will be possible to produce food containing mealworm on a mass scale.

Currently, EFSA is proceeding with eleven applications concerning insect species or certain products made from them. The following are in the risk assessment stage:

– Dried crickets (*Gryllodes sigillatus*), EFSA-Q-2018-00263;
– Whole and grinded lesser mealworm (*Alphitobius diaperinus*) larvae products, EFSA-Q-2018-00282;
– *Locusta migratoria*, EFSA-Q-2018-00513,
– *Acheta domesticus*, EFSA-Q-2018-00543,
– Mealworm (*Tenebrio Molitor*), EFSA-Q-2018-00746
– Whole and ground mealworms (*Tenebrio molitor*) larvae, EFSA-Q-2019-00101;
– Whole and ground grasshoppers (*Locusta migratoria*), EFSA-Q-2019-00115;
– Whole and ground crickets (*Acheta domesticus*), EFSA-Q-2019-00121;
– Defatted whole cricket (*Acheta domesticus)* powder, EFSA-Q-2019-00589;
– *Tenebrio molitor* (mealworm) flour, EFSA-Q-2019-00748;
– Dried *Acheta domesticus*, EFSA-Q-2020-00748 [18].

## 3. Description of Selected Insects

Most insects are characterized by a well-balanced nutritional profile that is determined by their development phase. Insects can be consumed as eggs, larvae, pupae or adults. The crude protein content ranges widely from 20% to 70% on a dry-matter basis. According to the collected data, the protein content in insects is higher than that in most plants, but it is also higher than that for most commercially produced meat, poultry and eggs [19,20]. Individual insect species may differ in the protein content, amino acid profile and fatty acid composition, depending on the breeding and feeding methods as well as on development phase [21]. Insect meat contains all essential amino acids. It is characterized by low contents of only methionine and cysteine, yet it is rich in lysine, tryptophan and threonine. A deficiency of one of them or all is present in diets based on highly processed products comprised mainly of cereal products such as wheat, rice, cassava and maize [22]. Moreover, the digestibility of insect protein is, on the average, 76–98%, and is higher than that for peanuts and lentils, and only slightly lower than that for beef or egg white [23]. According to numerous reports and analyses, many edible insects are rich in fat. At the larva and pupa stages of edible insects, the fat content is higher than that in an adult insect. The fat content in edible insects ranges from 10% to 50%. All edible insect species contain essential mono- and polyunsaturated fatty acids, particularly linoleic and linolenic acids which are essential for the prevention of cardiovascular diseases. Moreover, certain insects may provide more calories in the diet than soybeans, maize or beef [24]. Fatty acids in insects are similar to fatty acids in poultry and fish in terms of the degree of unsaturation [25]. The cholesterol level in insects ranges from low to, approximately, the levels found in other animals, depending on the species and the diet. Cholesterol is the most common sterol found in insects. The average cholesterol content in the lipid fraction amounts to approximately 3.6%. In addition to cholesterol, edible insects can contain campesterol, stigmasterol, *β*-sitosterol and other sterols. Edible insects are rich in protein and fat while containing small amounts of polysaccharides (approximately 1–10%). In addition, some of the insects with an exoskeleton contain significant amounts of chitin, which reduces the digestibility of insects (2.7–49.8 mg/kg fresh matter). Whole shelled insects intended for consumption are slightly less accepted than products of vertebrate origin, mainly due to the presence of chitin. Chitin is considered to be indigestible fiber, even though the enzyme chitinase is found in human gastric juice. It was found, however, that this enzyme could be inactive, particularly in Europeans [26,27]. The chitin content can also lead to miscalculating the protein content. The protein content is usually calculated from total nitrogen using the nitrogen-to-protein conversion factor (Kp) of 6.25. This factor inflates the protein content, due to the presence of nonprotein nitrogen in insects. Janssen et al. and Ritvanen proposed lower conversion factor around 5.0. The removal of chitin increases the quality of insect protein to a level comparable with that for products derived from vertebrates [28,29]. In addition, insects are a rich source of vitamins, particularly vitamins B12, B2, biologically active form of vitamins A and β-carotene as well as mineral compounds of calcium, zinc and iron [4]. The most promising edible insects which are likely to be accepted in Europe include insects of the order *Orthoptera*: grasshoppers, crickets, and locusts, as well as insects of the order *Coleoptera*: the mealworm and buffalo worm larvae. These insects have so far the richest research data covering well known breeding requirements and their nutrition value. Most of them have already passed successfully through consumer acceptance tests in European countries. Intensive marketing campaigns have been launched already, what gives hope for further positive change of consumers approach.

### 3.1. Grasshopper (Orthoptera)

Grasshoppers are a traditional product in the diet of inhabitants of Asian and southern African countries as well as of Mexico [30]. Grasshoppers, mainly adults, are traditionally eaten raw following the removal of their wings. Traditional methods of their processing include frying and sun drying [31]. According to research, the contents of ash, protein, fat, dietary fiber and carbohydrates differ significantly between various grasshopper species. The tested species were characterized by a protein content varying from 43.9% to 77.1%. The fat content ranged from 4.22% to 34.2%, while the dietary fiber content ranged from 3% to 12.17%. A study by Lehtovaara et al. demonstrated that the modification of food consumed by grasshoppers may affect their fatty acid profile. An increase in the contents of linoleic and α-linolenic acids, EPA and DHA in the food consumed by grasshoppers increases the contents of these fatty acids in grasshoppers. Such operations result in an improved ratio of n-6 to n-3 acids [32]. A small amount of carbohydrates (ranging widely from 0.001% to 22.64%) were observed in the analyzed insects. Grasshoppers were found to be rich in vitamins, mainly B1—0.59 mg/100 g, B2—from 0.27 to 0.87 mg/100 g, and B3 whose levels varied considerably from 1.56 to 3.97 mg/100 g of the product. Vitamin C content ranged from 23.8 to 25.5 mg/100 g of the product. The content of vitamin A as the retinol equivalent amounted to 16 mg/100 g, and the vitamin D content ranged from 4.12 to 21.3 μg/100 g of the product [30]. A study by Ademolu et al. analyzed the mineral content. Phosphorus was found in the highest amount (218 mg/g d.m.). The potassium content was at a level of 7.61 mg/g, and the sodium content was 3.06 mg/g. The iron level was at a level of 1.84 mg/g, magnesium at 0.39 mg/g and zinc at 0.17 mg/g. The calcium content was 1.82 mg/g d.m. [33]. The composition of grasshoppers may vary within a species. A study by Kinyuru et al. compared the composition of brown and green grasshoppers of the species *Ruspolia differens*. Statistically significant differences were noted as regards the contents of water, ash and fat. The differences in protein content were not statistically significant. Therefore, it can be considered that the protein content in grasshoppers within a single species remains relatively stable [31]. In terms of the interspecies composition and within the species, there is great variability. When addressing the nutritional value, the specific species and the development stage of an insect need to be considered and not average values for the entire group of these insects. This, however, does not change the fact that all species are a valuable source of protein and are a product with a high nutrient density. Grasshoppers in the egg stage are characterized by the lowest protein content, while those in the last development stage have the highest protein content (>59%) [34]. All development stages of the grasshopper are characterized by a high glutamic acid content, which ranges from 7.60% to 10.00% of the total amino acid pool and is thus the dominant exogenous amino acid in the composition. All development stages of the grasshopper contain 9 out of 10 essential exogenous amino acids. Subsequent stages of development exhibit an increase in the exogenous amino acid content, which results from the development of the exoskeleton structures. The limiting amino acid in the composition of grasshoppers is tryptophan, which is absent in all development stages. In other studies, tryptophan was found in a small amount (0.51 g/kg) of protein. In the flour obtained from adult grasshoppers, the dominant amino acids included threonine (204 g/kg of protein) and proline (156.61 g/kg of protein) [35]. With an increasing degree of grasshopper development, the content of fat-soluble vitamins (A, D, and E) increases, because the lipid content increases with subsequent development phases [34].

Flour obtained from grasshoppers also contains antinutritional substances, i.e., tannins, oxalates and phytates, which may contribute to a decrease in nutrient bioavailability. Not only is the nutritional value of grasshoppers indicated by the initial nutrient content but also by the losses of vitamins and minerals resulting from their processing. Thermal processing enables the extension of shelf-life. Currently, roasting, drying and storage at room temperature in a non-transparent vacuum packaging or a transparent plastic container can extend the shelf-life to 12 weeks. When the vacuum-packed product has been precooled, the storage life can be extended, while maintaining desirable sensory characteristics for up to 22 weeks [36]. Unfortunately, drying grasshoppers results in decreasing the contents of riboflavin, folic acid, niacin, pyridoxine, retinol, ascorbic acid and α-tocopherol, while drying fresh or roasted grasshoppers reduces the digestibility of protein by 2–5% [37,38].

Grasshoppers consumed in a traditional manner may, to a small extent, be acceptable as food in societies for which they are not part of traditional cuisine. Alternatively, it is possible to introduce them in a powdered form as an additive to conventional products. This, however, may result in decreased product palatability along with an increased powder content and decreased overall acceptability of the product by consumers [39].

### 3.2. Cricket (Orthoptera)

The house cricket (*Acheta domesticus* L.) is considered to be one of the most promising farmed insects due to its attractive nutritional profile. The potential nutritional value of insects of the cricket (*Acheta domesticus*) species, particularly in the human diet, has been known for a long time. Apart from providing a rich source of high-quality protein for human consumption, crickets offer several other advantages as a source of food for humans. They have a short life span, produce numerous offspring and can develop within a wide range of environmental conditions. The average protein content in farmed crickets ranges from 56.2 to 60.0% d.m., and in all cases, the number of exogenous amino acids exceeds the standards recommended by WHO. The vast majority of crickets contain palmitic and oleic acids as well as two fatty acids essential for humans, i.e., linoleic and α-linolenic acids, which accounts for 63–122 mg/g d.m. of fatty acids. In the cricket composition, considerable amounts of minerals and trace elements are noted, namely calcium (366–480 µg/g d.m.), copper (8.5–9.2 µg/g d.m.), iron (16.2–26.7 µg/g d.m.) and magnesium (255–306 µg/g d.m.) [40,41].

It has also been observed that the insect’s sex may affect the nutritional value and chemical composition. For the cricket, both sexes are rich in protein and lipids. However, females contain a significantly higher amount of lipids (18.3–21.7 vs. 12.9–16.1 g/100 g of dry matter, *p* = 0.0001) and lower amounts of proteins than males (61.2–64.9 vs. 66.3–69.6 g/100 g of dry matter, *p* = 0.0001). Males contain more chitin (*p* = 0.0015) and nitrogen chains (*p* = 0.0003) than females [42].

It appears that age can also determine the nutritional potential of the house cricket. A study by Kipkoech et al. [43] examined the effect of age in order to determine the optimal harvesting time for the possible use of crickets to improve the feeding of children in Kenya. The results of the study indicate that the best time for gathering farmed crickets is between the 9th and the 11th week when the protein and mineral contents are optimal. This shows the importance of identifying the optimal time for gathering insects for consumption.

Since crickets used in food usually are in the adult form, they also contain chitin which, from the nutritional perspective, is an indigestible ingredient. Chitin is a modified polysaccharide (poly-beta-1,4-N-acetylglucosamine) that contains nitrogen with a structure analogous to that of indigestible cellulose. However, it is increasingly considered to be an insoluble fibre with potential prebiotic properties which may have a positive effect on human health through the selective promotion of the growth of beneficial bacterial species in the intestines, yet this compound is not sufficiently recognized. A study by Stull et al. (2018) assessed the effect of consuming 25 g of crickets per day on the composition of the intestinal microflora, while observing safety and tolerability. The results showed that the consumption of crickets was tolerable and non-toxic at the dose tested. Cricket powder supported the growth of the probiotic bacterium *Bifidobacterium animalis*, which increased 5.7 times. Cricket consumption was also associated with a reduction in plasma TNF-α levels. These data suggest that consumption of crickets may improve gut health and reduce systemic inflammations. However, to confirm above, more research is needed to understand these effects and their underlying mechanisms. A study by Osimani et al. [44] cricket (*A. domesticus*) powder was added to wheat flour to obtain bread with increased nutritional value. Bread loaves were obtained from doughs made using various mixtures of wheat flour and cricket powder added at an amount of 10% or 30% (calculated as wheat flour). Compared to control breads produced from wheat flour, breads containing cricket powder exhibited a higher nutritional profile in terms of the fatty acid composition, high protein content and the presence of essential amino acids. Bread enriched with 10% cricket powder received positive acceptance from consumers. The collected data demonstrated the good suitability of cricket powder for the production of enriched bread, which was confirmed by a study by Burt et al. [45] who assessed the nutritional value and acceptability of muffins made using cricket flour (CF) as compared to muffins made using a universal flour (AP), in a group of *n* = 198 subjects. The satisfaction ratings did not differ significantly, but the results were significantly higher for the texture of cricket-based muffins. Unfortunately, considerably lower sensory attractiveness, as compared to the control sample, was indicated. Nevertheless, the high nutritional value and proper functional characteristics are encouraging. The aim of the study by González et al. [46] was to examine the potential use of insect flour as a protein-rich ingredient in bakery products. The study used inter alia flour from *A. domestica* which replaced 5% of wheat flour. The addition of insect flour affected rheological properties (absorbability and stability) of dough during mixing, which was characterized by lower water adsorption. Breads containing flour from *A. domestica* exhibited volume and texture parameters similar to those of wheat bakery products, but with a higher protein and fibre contents, which confirmed the suitability of insect flour for the production of bread with an increased nutritional value.

One of the studies determined Canadian consumer attitudes towards entomophagy and assessed the consumers’ perception of cricket-based protein powders. Prior to consuming cricket protein powder, the majority of study participants believed that insects were a balanced protein source, yet they also thought that their consumption was undesirable. However, after consuming cricket protein powder, the study participants were willing to buy cricket powder and were ready to recommend it to their friends [47]. Protein preparations are widely used in the Western world, therefore, the use of insect-based products can be the right way of development of this food industry branch. The use of insect protein preparations in gluten-free diets may also be an interesting trend. The elimination of gluten in bakery products is a technological challenge, as the lack of gluten results in bakery products with a poor gas retention capacity during rising, which can be minimized thanks to the use of non-gluten proteins combined with hydrocolloids and/or enzymes [48] used cricket flour to make gluten-free sourdough bread suitable for people with coeliac disease. Based on the results obtained by Kowalczewski et al., it can be concluded that the use of cricket powder to enrich gluten-free bread can not only improve the nutritional value, but also effectively delay the process of bread staling. The doughs were fermented by a variety of methods. The following were analyzed: the pH and growth of microorganisms, volatile compound, the protein profile and the antioxidant activity before and after baking in relation to a standard gluten-free dough. The results showed that the doughs enriched with crickets and standard doughs had similar fermentation processes. Enrichment with crickets provided the breads with a typical flavor profile characterized by a unique bouquet of volatile compounds, consisting of nonanoic acid, 2,4-nonadienal (E, E), 1-hexanol, 1-heptanol and 3-octene-2-one, expressed in varying amounts depending on the type of inoculum The antioxidant activity was significantly increased in cricket bakery products, which shows that powder from these insects provides producers with a substrate with a high nutritional protein value and antioxidant properties. Research into cricket powder in the context of gluten-free food was also carried out by da Rosa Machado et al. [49]. Powder from crickets (*Gryllus assimilis*) was subjected to analysis and compared with lentil and buckwheat flours. Cricket powder exhibited high water and oil retention capacity and microbiological properties suitable for human consumption. The results confirm that enrichment with cricket powder may result in the production of gluten-free bakery products with acceptable technological properties and high protein contents. Since the addition of cricket powder increases lipid contents, it is recommended that oil-free preparations should be used to obtain better nutritional and functional results.

Apart from the use of cricket flours, powders and pastes in the food industry, it is also possible to obtain high-quality hydrolysates. In a study by Hall et al. [50] whole crickets were hydrolyzed with alcalase at concentrations of 0.5%, 1.5% and 3% for 30 min, 60 min and 90 min. The following were assessed: the degree of hydrolysis, amino acid composition, solubility and the emulsifying and foaming properties. The solubility of protein hydrolysates improved. The emulsifying and foaming characteristics exhibited better functional properties, which indicates that cricket protein has potential to be a component of designed food and functional food, which is also reported by other authors [51,52,53].

### 3.3. Locust (Othoptera)

Insects of the *Acrididae* family are the most morphologically diverse group of the *Orthoptera* order, which includes more than 7500 different species. As regards the locust, those most popular in the countries of Africa, Middle East and Asia include the migratory locust (*Locusta migratoria*), the desert locust (*Schistocerca gregaria*) and *Schistocerca americana*. The advantages of using locusts in the production of food as a food additive include their potential sensory properties and rich nutritional composition.

The locust, similar to other insects from the family *Orthoptera*, is rich in protein, essential fatty acids and fibre. Data concerning the composition of locusts vary considerably and are determined by the species, habitat, the insects’ diet, the metamorphic stage and the processing method. It was noted that the average nutritional value of the edible locust is approximately 400–500 kcal/100 g of dry matter and 179 kcal/100 g of raw locusts [22]. The protein content ranges from 50% and 65% for the African migratory locust (*L. migratoria*). There are few data on the locust composition. Tests for the contents of crude protein, fat, carbohydrates and ash were conducted by Clarkson et al. [54].

Crude protein content in dry matter (50.79%) was similar to that in studies by Mohamed et al. [55] but considerably lower than that reported by [56,57,58]. The use of protein extraction from edible insects not only increases the protein content per 100 g and the digestibility of certain fractions (Yi, 2016), but may affect the acceptance of the product by consumers. Each fraction differs in yield, chemical composition, digestibility, color and functionality. Consequently, insoluble and soluble proteins have various potential applications as dietary components. The fat content (35%) was considerably higher than that reported by available sources [20], which shows the significant variation in the composition, depending on the factors determining the nutritional value. Oleic acid is the most commonly found fatty acid documented in the migratory locust species (37%) and is followed by palmitic acid (27.3%). The content of α-linolenic acid (15.7%) in the presented study fell within the range provided in the literature, i.e., 13.9% to 16.2% and the linoleic acid content in the presented study amounted to 8.9%. In addition, *L. migratoria* was characterized by the content of MUFA acids (38.49%), and PUFA acids (25.57%) [44].

Fat extraction is often a by-product of protein extraction, which results in the oil obtained from locusts being a good alternative source of lipids and food. The authors of another study emphasize that the omega-3 acid content in locust oil is an attractive characteristic for certain consumers, thus increasing the acceptance of an insect-based product [59].

Apart from minerals characteristic of all insects, locusts (*L. migratoria*) contain particularly large amounts of iron (8–20 mg/100 g d.m.), depending on their diet [22].

Since locusts are less popular on the Western market, few products contain protein extracted from these insects. When demonstrating, the promising potential of locusts as an alternative food or protein source, research indicates that consumers first need to accept this product in their diet [60].

A study by Purschke et al. [57] subjected pre-prepared migratory locust (*Locusta migratoria* L.) protein flour (MLPF) to enzymatic hydrolysis in order to examine the technical and functional properties of the product. The testing was conducted with the variability of the proteases used or their combination (the enzyme-substrate ratio) during the initial thermal processing (60–80 °C; 15–60 min) and hydrolysis (0–24 h). The study demonstrated that hydrolysis resulted in a higher emulsifying activity of 54% at a pH of 7, better foamability (326%) at a pH of 3 and better fat absorption capacity. The results of the study showed the potential of enzymatic degradation by improving the technological functionality of protein in the migratory locust. It may also be a promising approach to the reduction in the allergenic potential of insect proteins and, thus, to the formation of hypoallergenic products.

Insects can also be used as a milk equivalent. In a traditional food product, skimmed milk (SMB) in high-energy biscuits (HEB) was replaced with an alternative source of protein from powdered insects (silkworm pupa—SWP, and migratory locust pupa—LP). The authors of the study analyzed the physicochemical, sensory and microbiological properties of biscuits enriched with insects (LPB and SWPB) and compared them with skimmed milk (SMB) and nutritional standards USAID 2016 (STD). The LPB biscuits were characterized by a composition relatively similar to that of SMB, yet they were distinguished by twice as high contents of provitamin A (918.44 µg/100 g) and vitamin C (102.17 mg/100 g) than the recommended standards. The study demonstrated that high-energy biscuits enriched with edible insects obtained a surprisingly good rating of the sensory and microbiological assessment [61].

Another study compared biscuits prepared using insect oils and vegetable oils. The water extraction method was applied to obtain oils from two grasshopper species commonly consumed in Africa (the desert locust *Schistocerca gregaria* and the African nsenene *Ruspolia differens*). A dietary assessment was conducted, which demonstrated that biscuits prepared using *S. gregaria* oil exhibited a significantly higher crude protein content than other biscuits. A comparative analysis of the composition of oils isolated from two commonly consumed insect species showed that the oils were richer in omega-3 fatty acids, flavonoids and vitamin E than vegetable oils. The consumers’ acceptance was high for biscuits prepared using *R. differens* oil (95%) and sesame oil (89%) compared to biscuits with olive oil and *S. gregaria* oil. It is worth noting that the biscuits prepared with insect oils had more than 50% distaste in aroma and flavor. However, the results showed that the use of edible insect oils in biscuits encouraged consumers to taste food products of insect origin. In order to reduce the aftertaste in finished confectionery products, additional tests involving the use of refined or flavored insect oils need to be conducted [62].

The locust differs from ordinary grasshoppers in its ability to swarm over long distances, and is among the oldest migratory pests. In 2020, FAO recognized that the locust plague in Africa had been the most aggressive for 70 years. Many ideas for limiting this phenomenon have been put forward. One of them is an idea of using the locust (*Schistocerca gregaria*) on a mass scale as an alternative source in poor countries suffering from hunger [63]. It is difficult to judge whether locust-based products can be a source of food in Europe as well. It appears that it is the locust that has the slimmest chance to emerge on the European market because, as regards insects in general, it has more negative associations than other insects.

### 3.4. Mealworm (Coleoptera)

In the group of the most promising insect species intended for human and animal consumption, besides *Orthoptera*, there is a group of *Coleoptera* which includes the mealworm beetle (*Tenebrio molitor* L.) from the family *Tenebrionidae*. The duration of this insect’s development cycle is determined by environmental conditions. The life of the mealworm beetle comprises four stages: the egg (hatching after 3–9 days), larva (from 1 to 8 months), pupa (from 5 to 28 days), and the adult form (2–3 months). They tend to gather in warehouses where they attack and damage agricultural products stored there, mainly cereals and related products (flour, bran and pasta) [64,65].

The mealworm beetle is omnivorous, therefore, under breeding conditions, it can be fed with products of both animal and plant origin, with a daily ration containing at least 20% protein. Thanks to the opportunity to feed it with waste (in Europe, the use of only plant-based waste is permitted), the development of mealworm beetle breeding can contribute to decreasing the problem of disposing of a proportion of waste. Research also demonstrated that mealworm beetles are capable of biodegrading durable petroleum-based plastics, including polystyrene and polyethylene [66,67].

Insects intended for consumption are killed by freezing or heating. Then, due to the high moisture content (approximately 68%), they are dried, which allows them to be stored and transported more safely. The powder obtained from mealworm larvae, fed with cereal bran or flour, takes on a color ranging from light to medium brown. It is characterized by a sweet, almost nutty flavor and a nutty/cocoa aroma. Due to their high fat content (25–35%), dried insects are sensitive to oxidation, therefore, prior to grinding them, they are additionally subjected to a defattening process which ensures better product stability during the storage. In this way, flour is obtained which is used for feeding animals, including fish [68,69].

It is more effective to use the mealworm beetle in the larval form than in the adult form. This is due to the shorter breeding period, lower costs of obtaining the particular insect form and the greater amount of the material obtained. *T. molitor* larvae are very nutritious and are characterized by good flavor, digestibility and functionality [70,71]. These insects are easy to breed and exhibit a rather constant protein content. For this reason, they are farmed industrially as feed for pets, animals in zoological gardens, as well as for farmed animals (fish, swine, and poultry) and humans.

Depending on the farming conditions and the processing method, the nutritional value of mealworm larvae may vary. Crude protein content may range from 46.44% to 60.21%. Both the protein contained in the larvae and the amino acid profile are high quality. The amino acids with the highest contents include leucine (2.21–7.31%), lysine (1.58–5.76%) and valine (1.89–5.29%). The crude fat content ranges from 19.12% to 37.7%. The unsaturated fatty acid level is approximately 77–79%. Moreover, *T. molitor* larvae contain essential polyunsaturated fatty acids. The crude fibre content ranges from 4.19% to 22.35%, and the ash content from 2.56% to 6.70%. The amount of chitin, regarded as indigestible fibre, varies depending on the insect’s stage of life. In the larvae, the following minerals were determined: calcium (0.04–0.50%), phosphorus (0.70–1.04%), sodium (0.11–0.36%), potassium (0.74–0.95%), magnesium (0.20–1.63%), iron (63.00–100.02 mg/kg), zinc (102.00–117.40 mg/kg) and copper (12.30–20.00) [67].

Each substance and product authorized for consumption must meet the basic requirement of ensuring consumer safety. For this reason, each insect must be thoroughly checked for potentially hazardous components. Insects contain protective substances produced by exocrine glands. The mealworm beetle produces benzoquinones which are dangerous to both animals and humans. As the insect develops, this metabolite is accumulated [64].

In Europe, the mealworm beetle is regarded as rather disagreeable in taste. However, the use of insects added in the form of a powder as a component enriching the nutritional value of the product appears to be the most promising for food production. This is due to the convenient form of the product, which enables its precise dosage and may limit the neophobia phenomenon that occurs when whole insects are served. In Mexico, corn tortillas with the addition of mealworm larvae powder (1 g per 14 g of cornflour) were subjected to analysis. The study involved *n* = 18 participants whose task was to assess the flavor and texture. Due to the additives, the new product was accepted by respondents as it was characterized by a better structure and flavor than the control sample (made from corn flour only). The addition of mealworm beetle powder changed the tortilla color into a darker one, which did not lower the level of acceptability of the product subjected to testing [72]. This also offers hope for the development of production of this insect in Europe.

### 3.5. Buffalo Worms (Coleoptera)

Another insect that arouses interest is the litter beetle *Alphitobius diaperinus*, referred to as the buffalo worm, belonging to the order *Coleoptera* and the family *Tenebrionidae*. Adult individuals reach a length of 5.5–6.7 mm and have a wide, oval, shiny dark-brown or black body. Beetles are a group of insects which can be problematic in the human diet after reaching full maturity, which is contributed to by the presence of the wings, exoskeleton, limbs, etc. For that reason, as regards the buffalo worm, it is mainly the larvae that are used for further processing. Hormonally modified beetle varieties can be often used so that it can be followed by the pupal metamorphosis process taking place [73,74].

The buffalo worm is not as well researched an insect as, for example, the house cricket or the mealworm beetle, yet the available data show that it can be used primarily for the production of powder (flour) or in the freeze-dried form. The powder can be used as an addition to traditional flour or to produce high-protein functional products [74,75].

Buffalo worm larvae are characterized by a high nutritional value, particularly in view of their protein content and the amino acid composition as well as the contained fatty acids. Crude protein content ranges from 58.03 to 65 g/100 g d.m., while the fat content ranges from 13.4 to 29 g/100 g d.m. [76,77,78]. It should also be noted that, in a comparison of five species (*Tenebrio molitor*, *Zophobas morio*, *Alphitobius diaperinus*, *Acheta domesticus* and *Blaptica dubia*), the litter beetle was characterized by the highest content of exogenous amino acids (459 mg/g of crude protein): histidine—34 mg/g, isoleucine—43 mg/g, leucine—66 mg/g, lysine—61 mg/g, methionine + cysteine—26 mg/g, phenylalanine + tyrosine—120 mg/g, threonine—39 mg/g, tryptophan—12 mg/g, and valine—58 mg/g of crude protein. As regards fats, the SUFA content is 40.6 g/100 g d.m. (mainly C16:0 26.4 mg/100 g d.m.), the MUFA content is 37.8 mg/100 g d.m. (C18:1, *cis*–9 35.9 g/100 g d.m.), and the PUFA content is 21.69 g/100 g d.m. (C18:2, cis-9.12 20.29 g/100 g d.m.). In addition [76] determined certain functional properties of protein fraction 5, including protein foamability and gelling ability. As regards proteins from all insects, the foam stability was determined to be low irrespective of the pH value (3, 5, 7, 10). However, gels were already formed at 30% *w*/*v* and 15% *w*/*v* at the pH of 7 and 10. The protein derived from the buffalo worm formed the strongest gels, which indicates its potential functional properties [79].

Insects are referred to as a good source of minerals. According to data, the buffalo worm was characterized by the highest Fe and Zn contents of all farmed species [80]. The high bioavailability of iron has been confirmed by other studies. With so many applications and high nutritional value, the buffalo worm larvae may become a valuable ingredient that enriches our diet. However, obtaining consumer acceptance could be a significant barrier.

### 3.6. Silkworms

It appears that the above-described insect species cover the possibilities of the European market, even though certain opportunities are associated with the use of silkworms, caterpillars (*Lepidoptera*), honey bees, wasps and ants (*Hymenoptera*), termites (*Isoptera*), dragonflies (*Odonata*) and flies (*Diptera*) [81]. In the light of literature data, silkworms which are characterized by a very valuable composition appear to be very interesting in view of their numerous applications. The protein content is estimated at 20–21.6%, fat content at 17.5–19.9%, and the carbohydrate content at as much as 38.5–40.9%. Both the larvae and the pupae of *B. mori* are rich in important minerals such as (larva/pupa; mg/100 g): sodium (10.52/11.66), potassium (18.65/22.45), calcium (20.31/26.65), iron (5.31/6.33), magnesium (31.24/27.53) and zinc (35.63/37.5) [82]. The possibility of using ground silkworms as a component in the production of pasta was investigated. To this end, buckwheat flour, wheat gluten and silkworm powder which replaced 5% and 10% of buckwheat flour were used. With an increase in the addition of silkworm flour, the protein content in pasta increased (from 26.2 g/100 g to 30.3 g/100 g), while the carbohydrate content decreased (from 59.5 g/100 g to 54.9 g/100 g). Researchers also analyzed the results of organoleptic evaluation, which indicated that the addition of 10% of silkworm flour increased the general rating of an organoleptic assessment of the pasta in relation to the control sample. It was proven that enriching buckwheat pasta with silkworm powder may improve both the nutritional value and the consumer assessment results [83].

It should be noted, however, that *B. mori* contain antinutritional substances (larva/pupa), e.g., saponins (6.88/7%), alkaloids (8.55/8.61%), oxalates (0.91/1.22 mg/g) and phytates (72.89/110.16 mg/g). In addition, there are reports that draw attention to hazards associated with potential allergies to silkworm proteins [84]. It was found that since the known allergens contained in protein extracted from silkworm pupae, within the range from 25 to 33 kDa, were resistant to thermal, enzymatic and acid–alkali modifications, research into allergenicity should actually focus on these proteins [84]. In view of the insufficient number of studies, it appears that silkworms have little chance of emerging on the European market, all the more so as the level of consumer acceptance is even lower than for other insects concerned [85].

## 4. Insect Consumption Preferences in Europe

Nutritional neophobia occurs as an evolutionary adaptation aimed at avoiding potential hazards resulting from the consumption of novel foods. This situation affects various aspects of human nutritional behaviour, including nutritional preferences and choices [86]. The approach to edible insects is particularly negative for consumers in countries with no tradition of insect consumption. Insects arouse disgust and aversion [87]. It is worth stressing, however, that a significant number of edible insects are herbivores that feed on fresh leaves or wood. From this perspective, they are more hygienically safe than the seafood or frogs that are popular in Europe [88]. However, the barrier is culinary practice and the difficulty in the integration of insects into existing dietary practices. It seems that marketing efforts and attempts to combine insect products with traditional eating habits have not brought the expected results yet. The cultural, social, and psychological aspects in consumers may be crucial when they decide to try novel food of insect origin. Consumers are not certain of safety and pay attention to possible hazards associated with insect diseases and the conditions resulting from consuming them. It is noteworthy that Americans or Asians [89,90,91] are more inclined to introduce insects into their diet than the inhabitants of Europe [91,92,93] or Australia [94,95,96,97].

The decision to introduce insects into the diet, particularly in Europe, is linked to the understanding of the wider context: social, economic, and ecological. In routine consumer studies, it is the same determinants, i.e., the price, flavor, availability and habit, that usually determine the choice of a food product. However, as regards insect-based products, consumers are guided by different criteria. The main emphasis is placed on the aspect of the so-called higher necessity in the name of the common good. This establishes completely new tasks and expectations for producers and the market [98]. Table 1 provides current research into the preferences with regard to and acceptance of insects or insect-based products among the inhabitants of European countries.

In general, it needs to be stressed that the unwillingness to consume insects is mainly related to concerns about the flavor, aroma and structure of the product as well as health safety. The gathered insects are usually scalded with hot water following a starvation period of 1–3 days. Further culinary processing includes cooking, roasting, frying or drying. All additional technological operations result in changes to the flavor and aroma while offering the possibility of modifying them. Insects’ flavors are very diverse, which is supposedly due to the pheromones found on the insect body. The flavor can also be modelled using properly prepared feed and farming conditions as well as the thermal processing method. Roasted or fried insects are considered to be the tastiest. Consumers point out that the most common flavors include nutty, mushroom, forest, fish or baked potato flavor. In order to improve the acceptability on the European market, it is possible to purchase freeze-dried insects enriched with various flavorings and spices, for example, curry powder, garlic, paprika or fried onion flavor. The offer is not limited only to savoury flavors, as producers also offer insects in salty caramel or with chocolate. The color of a meal prepared from insects is of significance as well. Raw insects are usually dark-grey to grey, which, from the consumer’s perspective, is not an attractive feature. On the other hand, due to thermal processing, they take on a red color with shades of brown. Properly dried or freeze-dried insects take on a golden color [22].

The texture of insects ranges from crunchy to soft [105]. Some of them are very hard and have an irregular structure, which may considerably limit the placing on the market and the consumers’ acceptance. Insects with exoskeletons are crunchier due to the presence of chitin. On the other hand, larvae and caterpillars have a more delicate structure. The acquisition method and technological processing are of significance as well. In Europe, insects are most often sold in whole, freeze-dried or as a powder. It appears that the use of insect flour or protein concentrates as a food ingredient is by far most likely to be successful on the market [106] used the addition of insect protein hydrolysate in the production of sausages. Many positive functional characteristics were noted. Enrichment with insect flour decreased the moisture content in the sausage, which contributed to a change in rheological characteristics. Protein has repeatedly been the subject of research into the possibility for using it in bakery and confectionery production [69,103]. In one of the studies, grasshopper and mealworm beetle flours were added to traditional Turkish egg noodles. The assessed samples of egg noodles exhibited better functional effects, but the sensory assessment indicated lower acceptance towards the control sample. However, the rating was not disqualifying [107]. Insect proteins are also used as concentrates and isolates in designing functional food. Solubility is one of the major functional properties which regulate the food modelling processes. The degree of protein solubility in an aqueous solution determines its foaming, gelling and emulsifying abilities [23]. Having considered all functional characteristics of insect protein, they are recognized as distinguishable among other protein sources in food. What is more, the introduction of insect protein into designed food may prolong the feeling of satiety. This aspect is rarely addressed in such studies. Having considered the problem of world hunger, on the one hand, and the obesity epidemic on the other, it appears appropriate to carry out further research into the satiating properties of insect protein [108].

## 5. Hazards Related to the Production and Consumption of Insects in Europe

**Figure 1:**
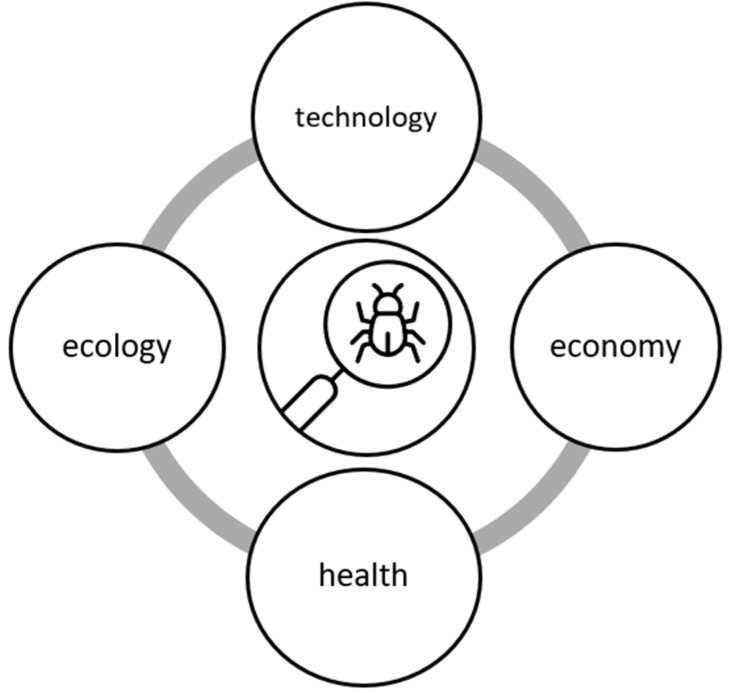
Gaps in the areas of knowledge concerning the edible insect market.

The rapidly developing industry involving insects as food is increasingly promoted as a sustainable alternative to other animal protein production systems. However, it is not completely clear if the European food market is ready for this type of food. The exact technological, economic, ecological and health-related advantages are not clear due to an overwhelming lack of knowledge on almost all of these aspects (Figure 1). It is essential to select appropriate species and the conditions for their growth, particularly as regards rooms, climatic factors and the entire control and surveillance system. It is necessary to examine whether or not the forced selection in one stage of an insect’s life has an adverse effect on other stages, for example through reducing the survival rate, reproductive functions or potential nutritional value. The system for controlling sick individuals and methods of their treatment, particularly the use of antibiotics and growth-promoting substances, is a gap in the knowledge. The system of insect feeding which includes the striving for breeding maximization while ensuring physical, biochemical and microbiological safety of insect-based food products, must also be subject to standardization.

From a technological perspective, not only the breeding process but also the method for preparing insects for the consumption, packing methods and effective distribution needs to be safeguarded. This, in turn, will determine the form of the sales system. The production of insects should be based on economic prerequisites of sustainable development. It should provide sufficient quantities of food of acceptable quality and appropriate efficiency, which, due to certain constraints, is extremely difficult. It is necessary to calculate the costs related to the production, breeding and transport. It appears that this can be one of the barriers to the introduction into the global and European food market. Nowadays, most industrial production is based on high-efficiency drying or freeze-drying processes, which considerably increase the production costs.

If sustainable environmental development is to be the paramount feature of the mass production of insects for the consumption, it is necessary to conduct research related to sustainable development criteria, which are directly linked to crucial aspects of industrial development [109]. First of all, breeding may directly affect the adjacent natural systems. What is particularly dangerous is the possibility of an uncontrolled, extensive spread of insects into areas where they are an endemic species or are not found in a particular ecosystem at all, which can have very serious consequences, both environmentally and economically. Moreover, there are no accurate data on the emissions of greenhouse gases released during insect production. It is indisputable that insect breeding on a mass scale generates fewer pollutants and residues than the breeding of other animals [26]. Moreover, the biomass conversion rate is lower and the production duration is much shorter than for any other animals. The use of water and land is lower than that for conventional breeding. Sometimes edible insects are crop pests and gathering them in the fields ensures both a source of food and lasting protection of crops without the use of chemical pesticides which must also be considered [110].

Another aspect is the thorough examination of the effect of insect consumption on health. As the subject of hazards to human health following the consumption of insects is new, there are few studies concerning this area of knowledge [109].

It follows from the available data that the emerging concerns can be considered in terms of chemical and microbiological hazards. The dynamic development of production raises questions about methods of killing insects and related ethical dilemmas. It will be necessary to develop a code and / or regulation setting morally accepted standards on insects’ welfare.

The most common chemical hazards include the presence of heavy metal and the residues of veterinary drugs, halogenated organic compounds and pesticides. Since the main passage of chemical exposure will be the substrate on which the insect grows, it is important to use a suitable substrate and ensure continuous monitoring during breeding [111]. Studies into insect heavy metal concentrations have mostly concerned insects bred in the feed industry and not as human nutrition products. Few studies report on increased levels of certain heavy metals such as cadmium, arsenic, lead and mercury [112,113,114]. This problem more often concerns insects gathered using a traditional method, where the natural environment of the particular area, in which the insects are found, is of crucial importance.

Moreover, problems resulting from the presence of toxins and veterinary drug residues were identified as well. Toxins contained in insects are most often the result of either the spontaneous synthesis of a natural toxin characteristic of the particular species or its accumulation, most often from a substrate. One of the studies analyzed 69 mycotoxins in flies. The study detected only three mycotoxins (enniatin A—12.5 µg/kg, A1—7.3 µg/kg, and beauvericin) [115,116]. It is believed, however, that the identified mycotoxin levels did not pose a health hazard, which was also confirmed by studies by [78,117].

The substrate quality is also linked to the presence of residues of veterinary drugs, mainly antibiotics, which could also pose an actual health hazard. Unfortunately, there is insufficient data on this subject [111]. Apart from drugs, agricultural waste residues, including pesticides and dioxins, can be hazardous as well, particularly when using a plant substrate [118].

Insects are a habitat of numerous microorganisms, including certain human pathogenic bacteria. Over the last few years, the focus has been on the microbiological safety of insects intended for consumption. It was assumed that the major hazard were zoonoses transmitted by insects. On the other hand, this is not supposed to happen under controlled breeding conditions. A greater hazard is posed by the microflora which may result from inappropriate breeding and the failure to comply with basic sanitary recommendations concerning processing and transport. Although it is believed that the viruses borne by insects are not dangerous to humans [119], mention a wide range of viruses that may pose a health hazard to humans.

Little is known about microbiology of processed insect products. One study examined a total of *n* = 38 samples subjected to various types of thermal processing. The presence of *Enterobacteriaceae, staphylococci*, *bacilli* as well as numerous yeasts and molds was detected. Even though each product type exhibited its own microbiological profile, the results for all samples were negative for the presence of *Salmonella*, *L. Monocytogenes*, *E. Coli* and *Staphylococcus aureus*, dried and powdered insects and dust particles contained *B. cereus*, coliform bacteria, *Serratia liquefaciens, Listeria ivanovii, Mucor* spp., *Aspergillus* spp., *Penicillium* spp. and *Cryptococcus neoformans.* Having compared the results with hygienic criteria for edible insects proposed by Belgium and the Netherlands, Class I products failed to meet many limits for bacterial count despite the absence of classical food pathogens. Therefore, it is recommended that Class I products should always be consumed following additional thermal processing [120].

Scarce studies show that the priority for microbiological purity includes the processing method and appropriate conditions for the storage of insects in each breeding farm [121]. Moreover, insects, just like all animals, can hide and transmit parasites, e.g., the nematodes *Gongylonema pulchrum* [122,123]. There is, however, insufficient data to determine whether such a hazard occurs under controlled industrial breeding conditions.

To sum up, the hazards to human health following the consumption of insect meat are largely induced by the quality of the breeding substrate and the proper implementation of all production stages, i.e., the processing, storage and distribution. Microbiological safety appears to be the biggest knowledge gap and that needs to be thoroughly investigated in the near future.

Edible insects are an important source of food worldwide. However, insufficient attention is paid to the undesirable allergic reactions caused by the consumption of insects, as insect protein is mentioned as a possible allergenic component [124]. Allergies to insect protein can be divided into the primary allergy to insects and susceptibility to cross-reactions with other allergens. There are few studies based on clinical trials on humans. Tests have been conducted on rats, mice and guinea pigs. The irritating agent was the proteins of the Japanese beetle, the mealworm beetle, and the cricket. Allergy to the mealworm beetle was only demonstrated on a mouse model. It was recognized that insect protein binds chitin and troponin, which may indicate that allergy to insects may also occur in humans [125]. A study by Francis et al. [126] suggests that exposure to insect allergy is not only oral but also includes inhalation or contact. Arginine kinase, paramyosin and chitin were responsible for allergic reactions in patients consuming silkworms. Similar study results were obtained by identifying the potential allergens in the mealworm beetle: arginine kinase, tropomyosin and both heavy and light myosin chain [127].

Certain researchers also indicate the possibility of cross allergies. One of the studies tested patients allergic to crustaceans and house dust mites. The entire test group, which exhibited allergy to crustaceans, exhibited allergy to mealworm beetle protein as well [128]. Leung et al. [129] reported a cross allergy between insects (grasshopper, cockroach, common fruit fly) and prawns for *n* = 9 subjects. In this case, tropomyosin was identified as the main allergen, probably because insects are closely related to crustaceans and HDM, in which the main allergens include tropomyosin and arginine kinase. Unfortunately, due to the scarce knowledge on this subject and the lack of diagnostic test consistency, it is not possible to clearly identify allergic relationships [130]. Additionally, the changes in insect proteins during thermal and further processing need to be examined [131]. As long as allergies to insects are poorly understood, it is necessary to be particularly careful and the information on packaging should include information on possible allergens. In addition, edible insects contain significant amounts of purines (adenine, guanine, xanthine, and hypoxanthine) and uric acid, which may limit the possibility of consumption in patients with gout [132].

## 6. Conclusions

Associating insects with food for humans triggers two completely different mental reactions. In countries where entomophagy is traditionally, or commonly, practised, insects are perceived as a valuable and traditional source of food, the knowledge of which is passed from generation to generation. Indeed, through globalization, insect consumption can sometimes be viewed, especially by younger people, as backward. On the other hand, in Western cultures, insects may provoke strong negative mental reactions, for example, repulsion.

In conclusion, the approach to entomophagy is determined by several major factors of a psychological, social, religious and anthropological nature. Since certain nutritional habits develop in childhood, it is suggested that in the future this will be the target group in highly developed countries.

Many supporters of the entomophagy sector believe that, in the years to come, a new emerging market of insects or their components (e.g., bakery products and snacks) may appear in many European countries, particularly in Northern Europe where certain insects had been available on the market even before the full application of the Regulation on new foods.

However, for such a trend to be sustained, it is necessary to understand the needs of consumers, therefore consumer acceptance is one of the most important challenges for food producers. Intensive marketing efforts and long-term educational strategies are needed to reduce uncertainty, ignorance and consumer reluctance and allow insects to be slowly introduced into the daily diet. In the case of European countries, it should be assumed that changes in eating habits in the context of the consumption of edible insects take time. Therefore, the method of small steps should be used here, in order to first target young Europeans who care about the environment and health, but who are also open and willing to change their eating habits. With the above in mind, an analysis of consumer preferences is required. The key to increasing interest in entomophagy is the development of products that are characterized by high trust, sensory appeal and health safety.

Future research should focus on finding the optimal conditions for breeding and processing insects into various forms with desirable functional properties and accepted sensory characteristics while maintaining a positive economic balance and environmental sustainability. One of the major challenges is the safety of consumption. This requires the development of precise legislation concerning production, distribution, sales and health safety. Therefore, further analysis should target these identified areas.

## Figures and Tables

**Table 1:** Summary of studies on implementation of insects as functional additives to food in Europe.

Kind of Insect	Reference	Research	Counrty	Results
(*T. molitor* L.) (*A. domesticus*) Insects flour Whole insects	[99]	insect chips, insect bar, whole insects	Italy *n* = 62	The highest palatability rating for a bar with insect meal (6.95), followed by whole crickets (6.64, crisps with insect meal (6.33). The lowest rating for insects in carmel (6.02).
(*A. domesticus*) Insects flour	[100]	Acceptability and sensory evaluation of energy bars and protein bars enriched with edible insect	Czech *n* = 96	The bars are acceptable to consumers in the Czech Republic, with the best rating for bars with the addition of a tropical flavor
(*A. domesticus*) Insects flour Whole inscets	[95]	Two types of jelly 1—with the addition of whole insects 2—with the addition of cricket flour	Italy *n* = 88	Insect jellies were rated better than before tasting. Jellies with the addition of cricket powder were better shaded than those with a visible insect.
(*T. molitor* L.) Insect flour	[69]	Addition of insect flour to bread dough in the amount of 5%, 10%	Italy *n* = 9	Bread with the addition of mealworm powder scored worse than the control sample. Bread with 5% insect flour was assessed slightly better
(*A. domesticus*) Cricket powder	[44]	Addition of powder to bread dough in the amount of 10%, 30%	Italy *n* = 9	Bread with the addition of cricket powder was evaluated worse than the control sample. Bread with 10% insect flour was rated slightly better
(*T. molitor*) Mealworm powder	[101]	50% addition to beef and green lentil burgers	Belgium *n* = 79	The mealworm burgers scored lower than the beef burger, but better than the lentil burger. The mixture of mealworm with beef was rated slightly better than with lentils.
(*T. molitor*) (*A. diaperinus*) Mealworm powder	[102]	Addition of insect powder to bread dough	Spain *n* = 327	Bread with the addition of mealworm powder was better rated than the bread with the addition of buffalo larvae powder and comparable to the control bread. The greater addition of mealworm powder (10%) made the bread with its addition the tastiest among the analyzed variants.
(*A. domesticus*) Cricket powder	[103]	Addition of 5%, 10%, 15% cricket powder to pasta	Poland *n* = 20	A consumer evaluation showed that the use of the CP additive was well received. The color of the pasta sample with 5% CP was described by consumers as resembling wholemeal pasta.
(*B. mori*) Silkworm powder	[83]	Addition of silkworm powder 5 and 10 g to buckwheat pasta	Hungary *n* = 98	The highest acceptance was obtained for pasta with a higher content of silkworm powder = 10 g
(*A. domesticus*) Cricket powder	[104]	Addition of cricket powder 5%, 10%, 15% to oat biscuits	Hungary *n* = 100	The biscuits with the addition of 5%/100 g CP obtained the highest acceptance, but the other variants also obtained the acceptance level

## Data Availability

Data available on request.
